# Neuronal mitochondria-targeted therapy for Alzheimer’s disease by systemic delivery of resveratrol using dual-modified novel biomimetic nanosystems

**DOI:** 10.1080/10717544.2020.1745328

**Published:** 2020-03-31

**Authors:** Yang Han, Xiaoyang Chu, Lin Cui, Shiyao Fu, Chunsheng Gao, Yi Li, Baoshan Sun

**Affiliations:** a School of Traditional Chinese Medicine, Shenyang Pharmaceutical University, Shenyang, PR China;; b Department of stomatology, The Fifth Medical Center of PLA General Hospital, Beijing, PR China;; c State Key Laboratory of Toxicology and Medical Countermeasures, Beijing Institute of Pharmacology and Toxicology, Beijing, PR China;; d School of Functional Food and Wine, Shenyang Pharmaceutical University, Shenyang, PR China;; e Instituto National de Investigação Agrária e Veterinária, I.P., Pólo Dois Portos, Dois Portos, Portugal

**Keywords:** Neuronal mitochondria, blood–brain barrier, biomimetic nanosystems, Alzheimer’s disease, resveratrol

## Abstract

Reactive oxygen species (ROS)-induced neuronal mitochondrial dysfunction is a key pathologic factor in sporadic Alzheimer’s disease (AD). Neuronal mitochondria have been proposed to be a promising therapeutic target for AD, especially for the failures of phase III clinical trials on conventional amyloid-β (Aβ) targeted therapy. However, the efficient intravenous delivery of therapeutic agents to neuronal mitochondria in the brain remains a major challenge due to the complicated physiological environment. Recently, biomaterials-based nanomedicine has been widely investigated for the treatment of AD. Herein, we devised a strategy for functional antioxidant delivery to neuronal mitochondria by loading antioxidants into red blood cell (RBC) membrane-coated nanostructured lipid carriers (NLC) bearing rabies virus glycoprotein (RVG29) and triphenylphosphine cation (TPP) molecules attached to the RBC membrane surface (RVG/TPP NPs@RBCm). With the advantage of suitable physicochemical properties of NLC and unique biological functions of the RBC membrane, RVG/TPP NPs@RBCm are stabilized and enabled sustained drug release, providing improved biocompatibility and long-term circulation. Under the synergistic effects of RVG29 and TPP, RVG/TPP NPs@RBCm can not only penetrate the blood–brain barrier (BBB) but also target neuron cells and further localize in the mitochondria. After encapsulating Resveratrol (RSV) as the model antioxidant, the data demonstrated that RVG/TPP-RSV NPs@RBCm can relieve AD symptoms by mitigating Aβ-related mitochondrial oxidative stress both *in vitro* and *in vivo*. The memory impairment in APP/PS1 mice is significantly improved following the systemic administration of RVG/TPP-RSV NPs@RBCm. In conclusion, intravenous neuronal mitochondria-targeted dual-modified novel biomimetic nanosystems are a promising therapeutic candidate for ROS-induced mitochondrial dysfunction in AD.

## Introduction

Alzheimer’s disease (AD) is becoming a severe socioeconomic problem in societies worldwide because of the fast-growing aging population (Ballard et al., 2011). Over the past decades, removing amyloid-β (Aβ) deposits has been viewed as ‘a promising therapeutic strategy’ based on the amyloid cascade hypothesis (Hardy & Allsop, 1991). Unfortunately, almost all the Aβ-targeting drug candidates have failed in clinical trials (Doody et al., 2013, 2014; Salloway et al., 2014). Recent clinical reports have found that Aβ deposits may also present in the brain with no clinical manifestations, and an elevated level of Aβ is not always consistent with sporadic AD (Jack et al., 2016). Many studies have further revealed that amyloidosis in sporadic AD may be secondary to other pathological processes (Selfridge et al., 2013; Caldwell et al., 2015). Therefore, it may be time to consider an alternative targeting approach for AD treatment.

Mitochondria are organelles for the oxidative metabolism of most eukaryotic cells. Reactive oxygen species (ROS) byproducts generated during the oxidative metabolism process can cause mitochondrial dysfunction, which involves the loss of mitochondrial biogenesis, defects in mitochondrial dynamics and mtDNA mutations (Lin & Beal, 2006; Yun & Finkel, 2014). Persistent ROS-induced neuronal mitochondrial dysfunction can lead to increased production and aggregation of Aβ, as well as the hyperphosphorylation of tau by activation of glycogen synthase kinase 3 (GSK3) (Lovell et al., 2005; Shafiei et al., 2017). When ROS-induced neuronal mitochondrial dysfunction exceeds the threshold, the characteristic pathological changes of AD, such as Aβ deposition and neurofibrillary tangles (NFTs) caused by the hyperphosphorylation of tau, occur (Yao et al., 2009; Wang & Chen, 2016). Furthermore, the deposition of Aβ and NFT damage the structure and function of mitochondria, and the vicious cycle causes AD progression (Kerr et al., 2017). Thus, ROS-induced neuronal mitochondrial dysfunction may be a key factor in the pathogenesis and progression of sporadic AD (Swerdlow & Khan, 2004).

Because Aβ may be an epiphenomenon of pathological development of sporadic AD rather than the cause of the disease, drug candidates that can protect neuronal mitochondria against oxidative stress from ROS would be of great value for sporadic AD treatment. For example, resveratrol (RSV), a natural polyphenol, has a variety of beneficial activities including antioxidant, anti-inflammatory, antiaging and neuroprotective effects, shown to relieve AD symptoms (Sawda et al., 2017). However, due to the physiological and pathological barriers in the brain, RSV is limited in clinical applications (Singh et al., 2008; Walle et al., 2004). It is difficult for traditional antioxidants to achieve the desired effect because of their limited distribution in neuronal mitochondria.

Currently, nanotechnology represents a promising approach for the delivery of therapeutic agents in mitochondria. An ideal nanocarrier used in the central nervous system (CNS) drug delivery should sufficiently cross the blood–brain barrier (BBB) while being efficiently specific to reach the targeted sites with suitable physiochemical features and good biocompatibility. However, it is a tremendous challenge to engineer all of the above characteristics into a single nanoparticle. For example, Kwon et al. developed a mitochondria-targeting ceria nanoparticle for AD therapy (Kwon et al., 2016). However, because these nanoparticles cannot cross the BBB, they are only administered via ipsilateral hippocampal stereotactic injection. Additionally, the nanoparticles are prepared using inorganic materials, which have several disadvantages, such as difficult biodegradation *in vivo*. Thus, the use of the designed nanocarriers for AD therapy in clinical applications may be limited.

New frontiers in the field of nanomedicine are advancing the research of novel biomimetic engineered delivery systems. Among them, red blood cell (RBC) membrane-coated nanocarriers (RBCm-nanocarriers) have received much research attention because of their advantages in drug delivery applications (Fang et al., 2017). Coating the RBC membrane on the surface of nanocarriers endows RBCm-nanocarriers with both suitable physicochemical properties of polymeric materials (e.g. complete biodegradation, sustained release, and fit to hydrophobic drugs) and unique biological functions of RBCs (e.g. prolonged systematic retention time, less reticuloendothelial system (RES) uptake, and reduced immunorecognition) (Chai et al., 2019). To enable the delivery systems to target the brain, many attempts have focused on the development of actively targeting RBC-nanocarriers that are modified with targeting ligands on the RBC membrane by the direct coupling or postinsertion method (Fang et al., 2013). However, the use of organic solvents and chemical reagents in the direct coupling process may compromise the protein profile of the RBC membrane, even influencing biological functions. As an alternative approach, the postinsertion method attaches the ligand (DSPE-mPEG-ligand) to the surface of the RBC membrane by incubation under mild conditions. To date, the most previous reported RBC-nanocarriers used PLGA nanoparticles as an ‘inner core’, which were prepared using organic solvents such as acetone, may lead to toxicity issues (Fuhrmann et al., 2015). Moreover, PLGA’s acidic by-products in degradation may render them inappropriate for extended use in in brain (Yang, 2010). Compared to PLGA nanoparticles, nanostructured lipid carrier based on natural lipids are more biocompatible, since they need not use of organic solvents during their fabrication and their degradation products may not influence the extracellular/intracellular environment (Fu et al., 2019).

**Figure 1.**
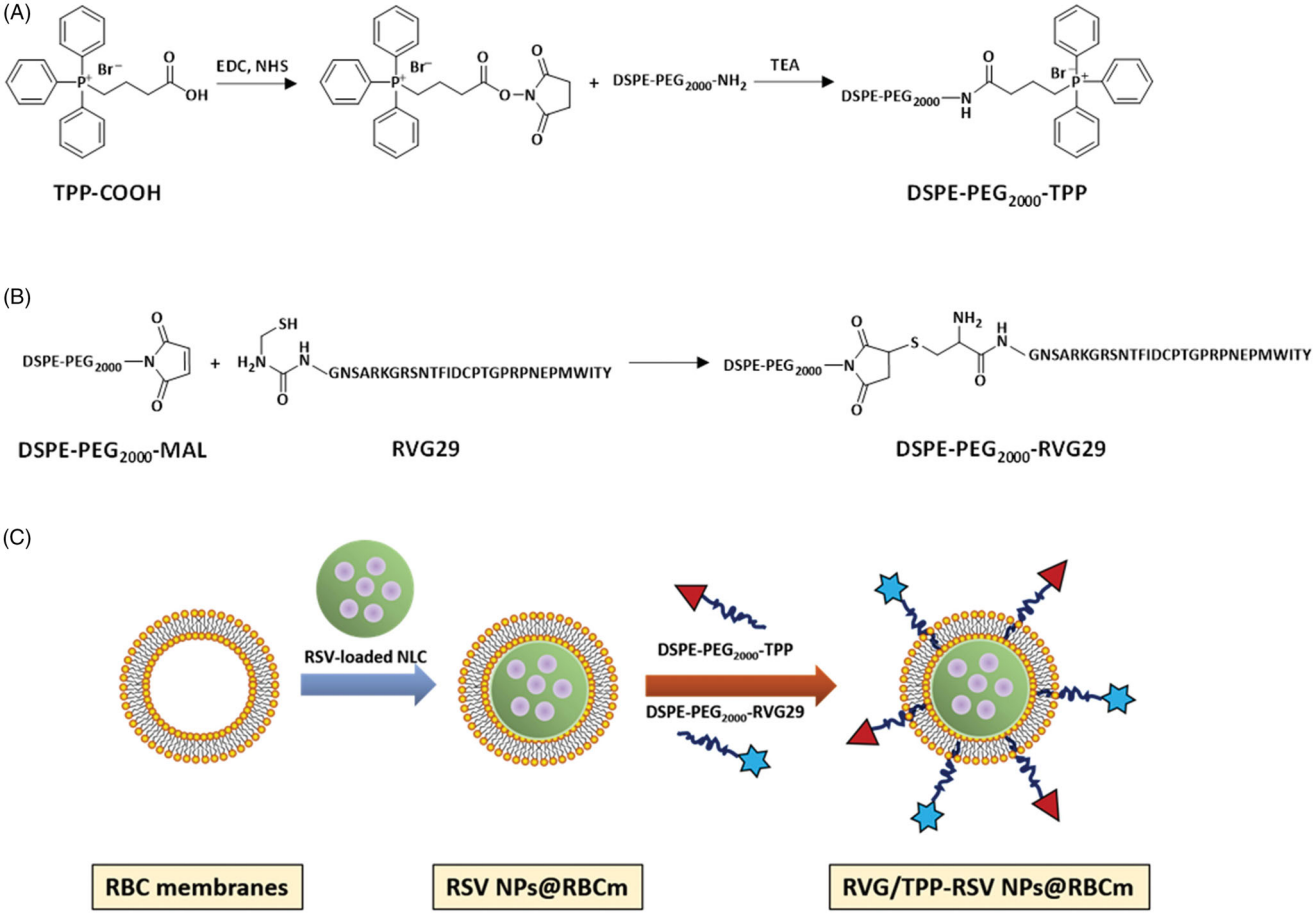
Preparation and characterization of dual-modified novel biomimetic nanosystems. Principle of the preparation of (A) DSPE-PEG_2000_-TPP and (B) DSPE-PEG_2000_-RVG29. (C) Schematic preparation of RVG/TPP-RSV NPs@RBCm. First, the RBC membranes were derived from RBCs and bare RSV-loaded NPs were prepared using an emulsification ultrasonication method. Next, the resulting RBC membranes were coated onto the surface of bare RSV-loaded NPs through mechanical extrusion to form RSV-loaded NPs@RBCm. Finally, DSPE-PEG_2000_-RVG29 and DSPE-PEG_2000_-TPP were inserted into the outer monolayer of RBC membranes to form RVG/TPP-RSV NPs@RBCm.

To augment the anti-AD therapy efficacy, in this study, we prepared RBC membrane-coated nanostructured lipid particles (NPs@RBCm) and functionalized them with dual targeting ligands for RSV delivery across the BBB to target neuronal mitochondria. RVG, a 29 amino acid peptide isolated from the coat protein of rabies virus, was applied as a promising candidate to improve nanocarrier’s ability to target the brain (Park et al., 2015; Cui et al., 2019). Triphenylphosphine (TPP) is one of the most promising mitochondria targeting ligands, which can be driven into mitochondria by taking advantage of negative mitochondrion membrane potential (Marrache & Dhar, 2012). On the basis of the aforementioned advances, we subsequently introduced RVG29 and TPP to the surface of the RBC membrane utilizing the lipid-insertion method. Schematic preparation of these dual-modified biomimetic nanoparticles (RVG/TPP-RSV NPs@RBCm) is shown in Figure 1(C). According to the navigation effects of RVG, the RVG/TPP NPs@RBCm accumulate in the neuron after crossing the BBB. With the aid of TPP, the RSV loaded biomimetic nanoparticles will further localize to mitochondria efficiently. Here, the physicochemical and biological characterization of the RVG/TPP NPs@RBCm were evaluated both *in vitro* and *in vivo*, and the *in vivo* therapy efficiency of the RVG/TPP NPs@RBCm for RSV was also explored in AD model mice.

## Experimental materials

### Materials

Amino-RVG29 and carboxy-TPP were provided by Xìan ruixi Biological Technology Co., Ltd (Xi’an, China). 1,2-Distearoyl-sn-glycero-3-phosphoethanolamine-N-[methoxy (polyethylene glycol) 2000]- maleimide (DSPE-PEG_2000_-Mal) and 1,2-Distearoyl-sn-glycero-3-phosphoethanolamine-N-[methoxy (polyethylene glycol) 2000]- ammonium salt (DSPE-PEG_2000_-NH_2_) were obtained from Avanti Polar Lipids, Inc. (Alabaster, AL, USA). Tween 80, cetyl palmitate, oleic acid, and cholesterol were kindly donated by Fenglijingqiu Commerce and Trade Co., Ltd. (Beijing, China). Resveratrol was purchased from Sigma-Aldrich (USA). Anti-CD47, anti-Iba1, anti-GFAP antibodies were purchased from Abcam (Cambridge, UK). MnSOD, GAPDH-specific antibodies and anti-rabbit antibodies conjugated to horseradish peroxidase were from Cell Signaling Technology (MA, USA). 1,1′-Dioctadecyl-3,3,3′,3′-tetramethylindotricarbocyanine iodide (DIR) and coumarin-6 (Cou6) were purchased from Biotium. All chemicals were of reagent grade and were obtained from Sigma-Aldrich, unless otherwise stated.

## Methods

### Cell culture and animal experimentation

In accordance with the previous report, mouse peritoneal macrophages (Schonhegrad & Holt, 1981) and primary astrocytes (Wang et al., 2015) were isolated from c57BL/6J mice. Brain microvascular cell line (bEnd.3) was obtained from ATCC (Maryland, USA). HT22 cells were purchased from the iCell Bioscience Inc (shanghai, China). HT22 and bEnd.3 cells were maintained in culture medium consisting of dulbecco’s modified eagle’s medium (DMEM) supplemented with 10% FBS, 100 IU/mL penicillin, and 100 mg/mL streptomycin. The cells were maintained in a 37 °C humidified incubator in a 5% CO_2_ atmosphere.

Male ICR mice (22–24 g) and Sparague-Dawley rats (190–210 g) were provided by Vital River Laboratories (Beijing, China). The APP/PS1 mice were obtained from Zhishan Healthcare Research Institute Ltd (Beijing, China). All animal experiments were complied with the code of ethics in research, training and testing of drugs issued by the Animal Care and Use Ethics Committee in Beijing Institute of Pharmacology and Toxicology.

### Synthesis of functional conjugates

DSPE-PEG_2000_-RVG29 was synthesized using an MAL-amino coupling reaction. Briefly, amino-RVG29 was dissolved in dimethylformamide (DMF) and added to DSPE-PEG_2000_-MAL, at an amino-RVG29 to lipid molar ratio of 1.5: 1. The mixture was gently stirred overnight at room temperature in the dark. The resulting supernatant was dialyzed against distilled water for 48 h. The purified dialysate was lyophilized and stored at −20 °C. DSPE-PEG_2000_-TPP was prepared using an NH_2_-carboxy coupling reaction. Briefly, carboxy-TPP was reacted with DSPE-PEG_2000_-NH_2_ (1.5: 1 molar ratio) in chloroform containing EDC (1-(3-dimethylaminopropyl)-3-ethylcarbodiimide hydrochloride) and NHS (N-hydroxysuccinimide) (5 eq.) at room temperature for 12 h with gentle mixing. The reactants were then dialyzed in a dialysis bag to remove the organic solvent and unconjugated reactants. The solution was lyophilized and stored at −20 °C. Finally, DSPE-PEG_2000_-TPP and DSPE-PEG_2000_-RVG29 were respectively analyzed by MALDI-TOF mass spectrometry (MALDI-TOF MS) and H^1^-NMR.

### RBC membrane derivation

The RBC membrane of ICR mice was collected using hypotonic treatment according to previously reported procedures (Dodge et al., 1963).

### Preparation of NPs

Normal nanostructured lipid carrier (NPs) were prepared by a modified emulsification ultrasonication method. Briefly, the lipid phase was produced by heating a mixture of cetyl palmitate, oleic acid, and cholesterol (3:1:1 weight ratio) at 80 °C to form a melted oil fraction. An amount of RSV (drug-to-lipid ratio= 0.1 w/w) or hydrophobic probe (DIR or Cou6, 100 μL) in a minute amount of ethanol was added to the melted oil fraction. The ethanol was removed by magnetic stirring for 20 min. The aqueous phase containing Tween 80 (0.7% w/w) was added into the lipid phase under magnetic stirring. The obtained primary emulsion was ultrasonicated and cooled until RSV-loaded NPs or hydrophobic probe-tagged NPs solidification dispersion was obtained.

The RSV encapsulation efficiency (EE) of the formulation was determined by minicolumn centrifugation using HPLC to quantify the RSV in the NPs (Careri et al., 2003) (EE%=(*W_total RSV_ − W_free RSV_*)/*W_total drug_*×100%; EE: 85.49 ± 1.36%).

### Preparation of novel biomimetic nanosystems

RBC membranes were sonicated for 6 min using a bath sonicator (KQ3200; Kunshan, China) at a frequency of 37 kHz and a power of 100 W. Next, the resulting RBC membrane vesicles were extruded repeatedly through 400-, 200-, and 100-nm polycarbonate porous membranes using a mini extruder (Avanti Polar Lipids, AL, USA).

To prepare plain NPs@RBCm (NPs@RBCm without a targeting ligand), 1 mL of RSV-loaded NPs or hydrophobic probe-tagged NPs at 5 mg^.^mL^−1^ were mixed with 1 mL of RBC membrane vesicles, followed by extrusion through a 100-nm polycarbonate membrane at least 5 times to obtain RSV NPs@RBCm or hydrophobic probe-tagged NPs@RBCm. Additionally, NPs@RBCm with RVG29 modification (RVG NPs@RBCm), NPs@RBCm with TPP modification (TPP NPs@RBCm) and those with both RVG29 and TPP modifications (RVG/TPP NPs@RBCm) were formed by the postinsertion method. Briefly, a lipid film of DSPE-PEG_2000_-RVG29 or DSPE-PEG_2000_-TPP was prepared by rotary evaporation and was further dried under vacuum for 24 h. The dried lipid film was subsequently hydrated with PBS (pH 7.4) to the formation of micelles at 37 °C. For the RVG NPs@RBCm or TPP NPs@RBCm preparations, a micelle solution of DSPE-PEG_2000_-RVG29 or DSPE-PEG_2000_-TPP was added into preformed NPs@RBCm and was incubated for 4 h at 37 °C in PBS (pH 7.4), respectively. To determine the optimal amount of specific DSPE-mPEG-ligand in the formulations, ratios of 1%, 3%, 5% and 7% were compared. For the RVG/TPP NPs@RBCm preparation, based on the screening results, certain amounts of DSPE-PEG_2000_-RVG and DSPE-PEG_2000_-TPP were incubated with preformed NPs@RBCm using the above conditions.

### Characterization of novel biomimetic nanosystems

The mean diameter and particle distribution of the prepared formulations were measured by dynamic light scattering (DLS) (Litesizer 500, Anton Parr, Austria). The morphology of RVG/TPP NPs@RBCm was characterized via transmission electron microscopy (TEM) (HITACHI, H-7650, Japan). The stability of RVG/TPP NPs@RBCm in 10% FBS in DMEM was evaluated using a Turbiscan Lab^®^ Expert (Formulaction, L’Union, France). The analysis of stability was carried out using the instrument software as a variation of back-scattering (Delta Transmission) profiles.

### Western blot

Western blot analysis was performed as described previously (Wang et al., 2010). Briefly, the collected samples of NPs, cell pellets or brain tissues were lysed in cold T-PER buffer containing 1% phosphatase inhibitors and complete mini cocktail (Roche, Switzerland). Protein concentrations were measured by the Bicinchoninic (BCA) assay kit (Pierce, China). Twenty micrograms of each protein sample were resolved by sodium dodecyl sulfate-polyacrylamide gel electrophoresis (SDS-PAGE). Gels were then transferred to polyvinylidene difluoride (PVDF) membranes (Millipore Corporation, USA). Transblotted PVDF membranes were blocked with 5% BSA for 1 h and then incubated overnight with the indicated primary antibody in 1% BSA at 4 °C and followed by the secondary antibody conjugated with horseradish peroxidase. Blots were visualized using ECL-Plus according to the manufacturer’s instructions.

### *In vitro* release profile

Dialysis bags (MW. Cutoff: 14 kDa) with 1 mL of RSV-loaded formulations were directly immersed into 30 mL of PBS (0.1 M, pH 7.4). At preset time points, 800-μL aliquots were withdrawn from the solution and the same volume of PBS was added. The RSV in the obtained samples was measured using HPLC as reported previously (Careri et al., 2003).

### Antiphagocytosis ability of novel biomimetic nanosystems

Mouse peritoneal macrophages (2 × 10^5^ cells/well) were seeded into a Petri dish in DMEM with 10% FBS at 37 °C in a 5% CO_2_ atmosphere. 4 h later, cells were washed to remove non-adherent cells and the culture medium was renewed. After 24 h, the cells were incubated with 5 μM Cou6-tagged NPs (bare Cou6 NPs, Cou6 NPs@RBCm, TPP-Cou6 NPs@RBCm, RVG-Cou6 NPs@RBCm and RVG/TPP-Cou6 NPs@RBCm) for 4 h. After incubation, the cells were collected and analyzed via flow cytometry (FCM) (BD FACSCalibur, USA).

### Cytotoxicity of blank novel biomimetic nanosystems

HT22 or bEnd.3 cells were seeded into 96-well plates (1 × 10^5^ cells/well) in DMEM at 37 °C in a 5% CO_2_ atmosphere, respectively. After incubation for 24 h, the cells were treated with different blank formulations and were incubated for another 24 h. At the end of the incubation, cell viability was evaluated using the MTT method.

### Hemolysis assay of blank novel biomimetic nanosystems

Two hundred microliters of red blood cells from healthy mice were seeded into 96-well plates. Next, the cells were incubated with PBS or various blank formulations. After incubation for 6 h at 37 °C, the cells were centrifuged and then the supernatants of the sample were measured at 414 nm using a microplate reader (Elx-800; Bio-Tek Instruments, USA).

### Cellular uptake and colocalization into the mitochondria

HT22 cells were seeded into a Petri dish at a density of 2 × 10^5^ cells/well overnight. After incubation, different 5 μM Cou6-tagged formulations were introduced. Live cells were harvested and resuspended in PBS for cellular uptake analysis via flow cytometry (FCM) (BD FACSCalibur, USA). For mitochondrial colocalization analysis, the Cou6-tagged formulations-treated cells were then stained with 0.5 μM MitoTracker Red (Molecular Probes, USA), followed by washing and keeping in pre-warmed PBS. Finally, cells were analyzed by confocal laser scanning microscopy (CLSM) (UltraVIEW Vox, PerkinElmer, USA) as soon as possible.

### Mitochondrial and intracellular ROS measurement

HT22 cells were cultured in 6-well plates (1 × 10^5^ cells/well) for 24 h. After incubation, cells were treated with different RSV-loaded formulations for 4 h and then co-cultured with Aβ_25–35_ (at a final concentration of 30 μM) for another 20 h. The final concentration of each RSV was 5 μg^.^mL^−1^. The relative levels of mitochondrial (·O_2_^−^ in mitochondria) and intracellular (H_2_O_2_) ROS were respectively measured using MitoSOX (Molecular Probes, USA) and CM-H_2_DCFDA (Molecular Probes, USA) as fluorescent probes via flow cytometry (FCM) (BD FACSCalibur, USA).

### *In vitro* oxidative stress-related biomarker assay

HT22 cells were treated with RSV-loaded formulations and co-cultured with Aβ_25-35_ as the method described above. After incubation, the cells were harvested for levels of Manganese superoxide dismutase (MnSOD) and malonaldehyde (MDA) measurements by western blotting and MDA assay kits (Beyotime Institute of Biotechnology, Jiangsu, China), respectively.

### Cell apoptosis assay

HT22 cells were treated with RSV-loaded formulations and co-cultured with Aβ_25–35_ as the method described above. Following 24 h of incubation, the cells were collected and stained using the Annexin V-FITC/PI apoptosis detection kit (Solarbio Science & Technology Co., Ltd, Beijing, China) according to the manufacturer’s instructions and were immediately analyzed by FCM.

### Transport across BBB and targeting of neuron cells

A BBB-HT22 cells co-cultured model was established according to previous reports (Omidi et al., 2003), with minor modifications. Briefly, bEnd.3 cells were seeded onto the upper chamber of the Transwell (Corning, NY, USA) and astrocytes were added to the underside of the Transwell at 37 °C and 5% CO_2_. After incubation for 5 days, a tight monolayer (transendothelial electrical resistance (TEER) exceeds 200 Ω^.^cm) was formed, HT22 cells were seeded in the bottom chamber, and then different 5 μM Cou6-tagged formulations were added to the apical compartment. After incubation for 12 h, fluorescent signals of the HT22 cells in the lower chamber were observed via FCM.

### Pharmacokinetic studies

The pharmacokinetic profiles of RSV-loaded different novel biomimetic nanosystems were measured in SD rats with a single dose of 5 mg^.^kg^−1^ RSV by tail vein injection. Additionally, free RSV diluted in dimethylacetamide, PEG_400_ and 5% dextrose solution (15: 45: 40 volume ratio) was injected into the other group of rats for pharmacokinetic comparison, as reported in previous studies (He et al., 2006). Blood was sampled from the retro-orbital sinus at different time points. The extraction of RSV was carried out as described previously (He et al., 2006). The RSV in the obtained samples was measured via liquid chromatography-tandem mass spectrometry (LC-MS/MS) as described previously (Wang et al., 2002). DAS 2.0 software was used to model the data of the experiment.

### *In vivo* imaging

Near-infrared dye DIR was applied as the fluorescence probe to evaluate the brain targeting efficiency of biomimetic nanosystems to mice. These mice were administered different DIR-tagged formulations via tail vein injection. Thirty minutes after i.v. injection, *in vivo* imaging was performed using IVIS Lumina II (Caliper Life Science, USA).

### Morris water maze (MWM) behavioral test

The APP/PS1 mice were randomly divided into five groups (20 mice in each group) and were treated with saline (control), free RSV and various novel biomimetic nanosystems carrying RSV by i.v. injection every two days for a total of 30 days at a concentration of 2 mg^.^kg^−1^ of RSV. Next, after the last administration, the mice were trained and tested in the Morris water maze (MWM) as described previously (Yao et al., 2016). Briefly, the mice were trained three times a day for 5 days. Finally, on the sixth day, with the platform removed, the mice were placed into the tank from the same fixed positions and were allowed to swim freely for 90 s with the times of platform crossing recorded. The mouse trajectory and escape latency were recorded using a computer-controlled tracking system (Shanghai Jiliang Software Technology Co., Ltd.).

### Enzyme-linked immunosorbent assay (ELISA) for Aβ measurements in brain

After MWM behavioral test, APP/PS1 mice were sacrificed. Brain tissues were lysed in buffer containing 50 mM Tris (pH 7.4), 150 mM NaCl, 0.5% sodium deoxycholate, RIPH, EDTA, leupeptin and protease inhibitors. The homogenate was centrifuged (100,000 g, 1 h, 4 °C), and the supernatant was stored at −80 °C for further analysis of soluble Aβ. The pellet was sonicated with 5 M tris buffer. The samples were incubated for 30 min at room temperature, then centrifuged (100,000 g, 1 h, 4 °C) and the supernatant was stored for analysis of insoluble Aβ. Collected samples were measured via Mouse amyloid beta peptide 1-42 ELISA Kit (Sangon Biotech Co., Ltd., Shanghai, China) according to the manufacturer’s instructions.

### Oxidative stress-related biomarker assay in the hippocampus

After MWM behavioral test, APP/PS1 mice were sacrificed. The brain hippocampus tissues were collected and lysed in buffer as mentioned above. MnSOD levels in hippocampal area were measured via western blotting method as mentioned above**.** MDA levels were determined by MDA assay kits.

### Inflammation-related biomarker assay in the hippocampus

After MWM behavioral test, APP/PS1 mice were sacrificed. The brain hippocampus tissues were collected and lysed in buffer as mentioned above. GFAP and Iba-1 levels were measured via western blotting method as mentioned above**.**

### *In vivo* safety evaluation

The healthy mice were randomly divided into six groups (*n* = 6) and were treated with saline (control), free RSV, various RSV-loaded formulations at a single RSV dose of 5 mg^.^kg^−1^ body weight via the tail vein, respectively. The daily body weight was recorded every day. At day 14, these mice were sacrificed, and samples of the heart, liver, spleen, lung, kidney and brain were collected and fixed in 10% formalin, embedded in paraffin, and sectioned at 8-μm thickness. The sections were then mounted on slides and stained with hematoxylin and eosin (HE).

### Statistical analysis

The data are presented as the means ± standard deviation (SD). The difference between any two groups is determined via ANOVA. *p* < .05 was considered to be statistically significant.

## Results and discussion

### Preparation and characterization of nanocarriers

**Figure 2.**
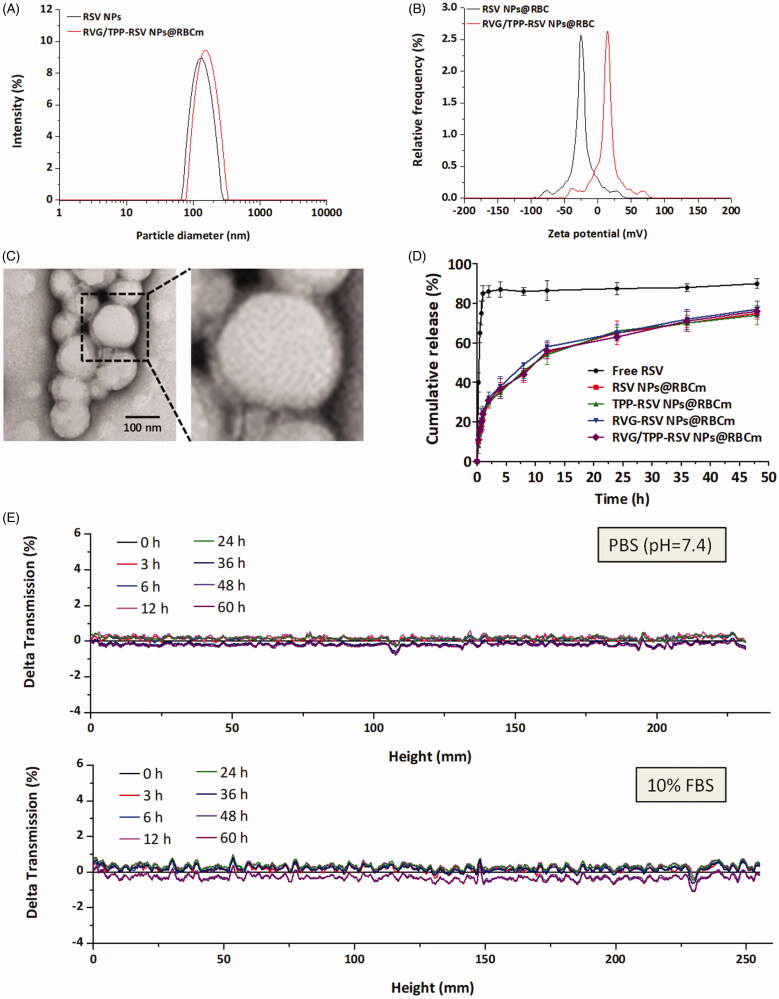
Physicochemical characterization of RVG/TPP-RSV NPs@RBCm. (A) Particle size and (B) zeta potential distribution of RVG/TPP-RSV NPs@RBCm. (C) Morphological appearance of RVG/TPP-RSV NPs@RBCm based on TEM. (D) In vitro release of RSV from RSV-loaded formulations at pH 7.4 at 37 °C. The data are presented as the means ± SD (*n* = 3). (E) Stabilities of RVG/TPP NPs@RBCm respectively in PBS and DMEM containing 10% FBS at 37 °C for 60 h. The transmission profiles were measured at each time point using a Turbiscan Lab^®^ Expert analyzer.

The preparation scheme for RVG/TPP-RSV NPs@RBCm is illustrated in Figure 1(C). First, the RBC membrane (‘outer shell’ of novel biomimetic nanosystems) was derived from RBCs and normal nanostructured lipid particles (NPs) (‘inner core’ of novel biomimetic nanosystems) were prepared using an emulsification ultrasonication method. Next, the resulting RBC membranes were coated onto the surface of NPs through mechanical extrusion to form RBC membrane-coated nanostructured lipid particles (NPs@RBCm) with a characteristic core-shell structure (Figure 2(C)). To improve targeting efficiency, the NPs@RBCm were modified using two synthesized functional conjugates, DSPE-PEG_2000_-RVG29 and DSPE-PEG_2000_-TPP. We conjugated amino-RVG29 to the distal end of DSPE-PEG_2000_-MAL by the reaction of amino groups with the active group MAL (Figure 1(B)). Similarly, DSPE-PEG_2000_-TPP was synthesized by the reaction of carboxyl groups on TPP with the active group NH_2_ contained in DSPE-PEG_2000_-NH_2_ (Figure 1(A)). The purified materials were obtained by dialysis (MWCO 3.5 kDa) against distilled water. Successful synthesis was evidenced by the band coincidence in H^1^-NMR (Supplementary Figure S1(B)) and the molecular shifts in MALDI-TOF MS analysis (Supplementary Figure S1(A)).

As an amphiphilic molecule, DSPE-PEG_2000_-RVG29/DSPE-PEG_2000_-TPP could be easily inserted into the outer monolayer of RBC membranes to form RVG/TPP NPs@RBCm by incubation under mild conditions. Based on the ligand density optimized results (data not shown), 7.5% DSPE-PEG_2000_-RVG29 and 5% DSPE-PEG_2000_-TPP in formulations were chosen for subsequent studies.

For a nanocarrier, the particle size and zeta-potential are crucial factors that determine the fate of nanocarriers. All four formulations showed an even distribution in size less than 160 nm (Figure 2(A) and Supplementary Table S1). This particle size was suitable for the particles in blood to cross into the tissue, approach cell surface receptors and facilitate intracellular transport (Zhao et al., 2012). As shown in Figure 2(C), the TEM images of RVG/TPP NPs@RBCm demonstrated that the particle sizes were close to those measured using the laser particle analyzer. The zeta potentials of ligand-modified novel biomimetic nanosystems (TPP NPs@RBCm and RVG/TPP NPs@RBCm) was higher than that of NPs@RBCm (Figure 2(B) and Supplementary Table S1), owing to the positive charge of the TPP. This would be beneficial for the internalization of ligand-modified novel biomimetic nanosystems because the cell membrane and mitochondrial membrane are negatively charged. Because stability is a prerequisite for further applications *in vivo*, the agglomerations of novel biomimetic nanosystems in PBS and 10% FBS in DMEM were respectively evaluated using Turbiscan Lab^®^ Expert at 37 °C to mimic *in vivo* conditions. According to this method (Celia et al., 2009), the transmission shown in Figure 2(E) was less than 2%. As shown in Supplementary Figure S3(A) and S3(B), there were no significant differences in both particle size and zeta potential of RVG/TPP NPs@RBCm within 60 h. The morphologies of particles by TEM shown in Supplementary Figure S3(C) has no obvious changes neither. The results indicated that the formulation exhibited good stability. The *in vitro* RSV release study was also performed to examine the drug release property of the novel biomimetic nanosystems. As shown in Figure 2(D), the in vitro drug release of RSV-loaded formulations was also carried out to characterize the RSV release properties. Compared to the rapid release of free RSV, RSV NPs@RBCm demonstrated sustained release behaviors with no initial release burst detected. In addition, similar release patterns were observed in the tested four formulations at every time point, implying that ligand modifications did not significantly affect the release behavior of RSV. Additionally, similar physicochemical characteristics of these formulations allowed us to specifically compare the effects of ligand modification on the anti-AD abilities of the novel biomimetic nanosystems.

### Preliminary safety test

**Figure 3.**
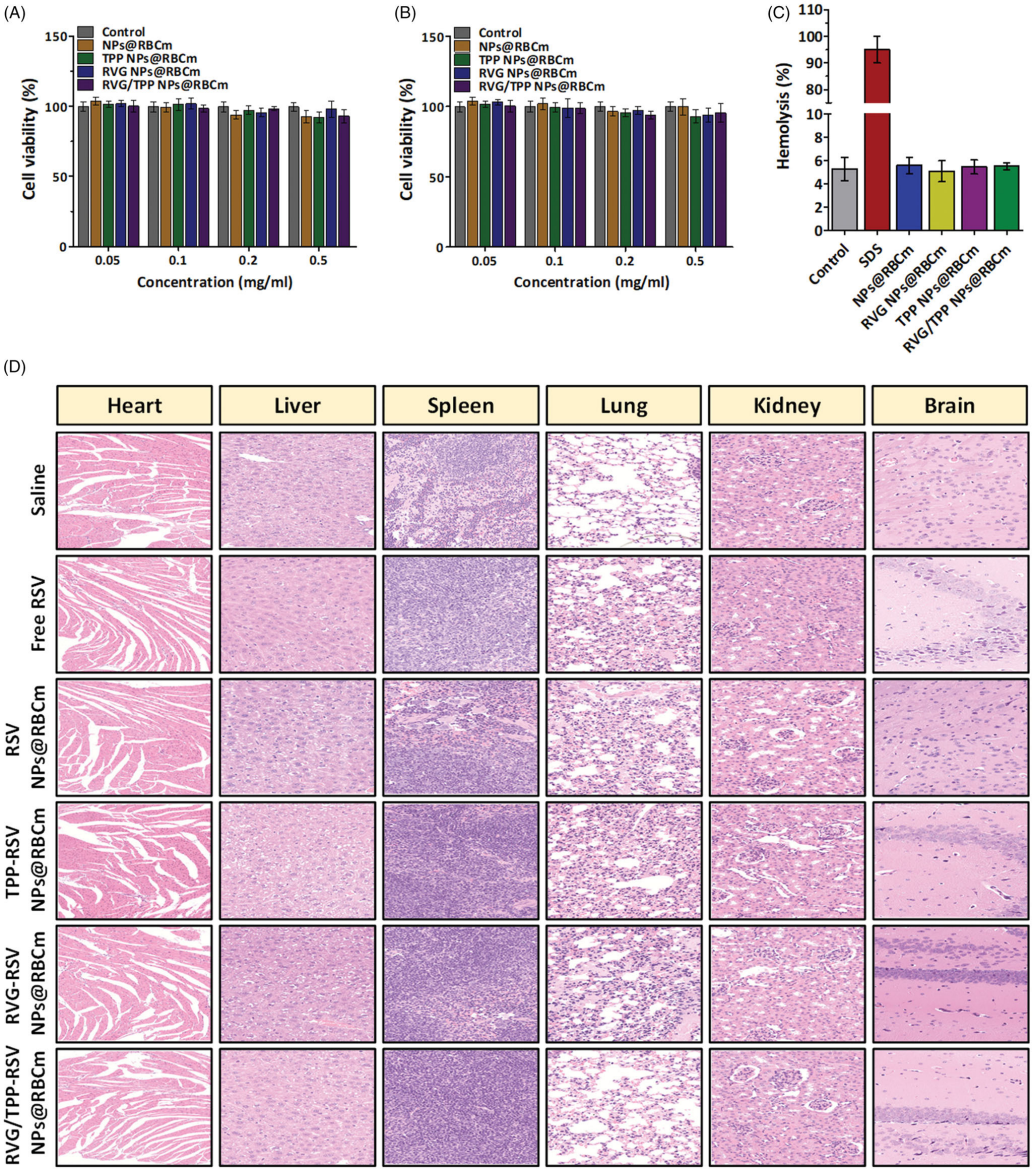
Preliminary safety evaluation of novel biomimetic nanosystems. Cell viability of (A) HT22 cells and (B) bEnd.3 cells incubated for 24 h with different concentrations of empty formulations. (C) Hemolysis assay of empty formulations. SDS as positive control. The data are presented as the means ± SD (*n* = 3). (D) Histological staining of organs from healthy mice treated with different RSV-loaded formulations.

In addition to having suitable physiochemical properties, an ideal nanocarrier should have minimal toxicity and high biocompatibility. We first conducted MTT and hemolysis assays on the various empty novel biomimetic nanosystems to assess their biocompatibility. As shown in Figure 3(A,B), the viabilities in both the HT22 cells and bEnd.3 cells of each group were higher than 90% after incubation with different empty novel biomimetic nanosystems for 24 h, revealing that these novel biomimetic nanosystems were relatively safe. Additionally, the percentage of hemolysis showed no significant change after treatment with various empty novel biomimetic nanosystems compared with that of the control, indicating that these novel biomimetic nanosystems are highly blood compatible (Figure 3(C)).

For *in vivo* safety assessment, no obvious abnormal state was observed among the tested mice within 14 days. Furthermore, on day 14, tissue sections from the heart, liver, spleen, lung, kidney and brain were assayed via HE staining after intravenous administration using different samples in healthy mice. As shown in Figure 3(D), compared with the saline (control) group, no indicators of damage were observed for these organs after treatment, suggesting that free RSV, and RSV NPs@RBCm did not cause systemic toxicity by i.v. injection at the current RSV dosage. Moreover, similar results were found in the above tests among RSV NPs@RBCm, TPP-RSV NPs@RBCm, RVG-RSV NPs@RBCm and RVG/TPP-RSV NPs@RBCm, suggesting that ligand modifications did not significantly affect the biocompatibility of novel biomimetic nanosystems. Taken together, these results confirmed the low toxicity and good biocompatibility of RVG/TPP-RSV NPs@RBCm is possible due to biodegradation of the ‘inner core’ and endogenous property of the ‘outer shell’.

### Long circulation feature

**Figure 4.**
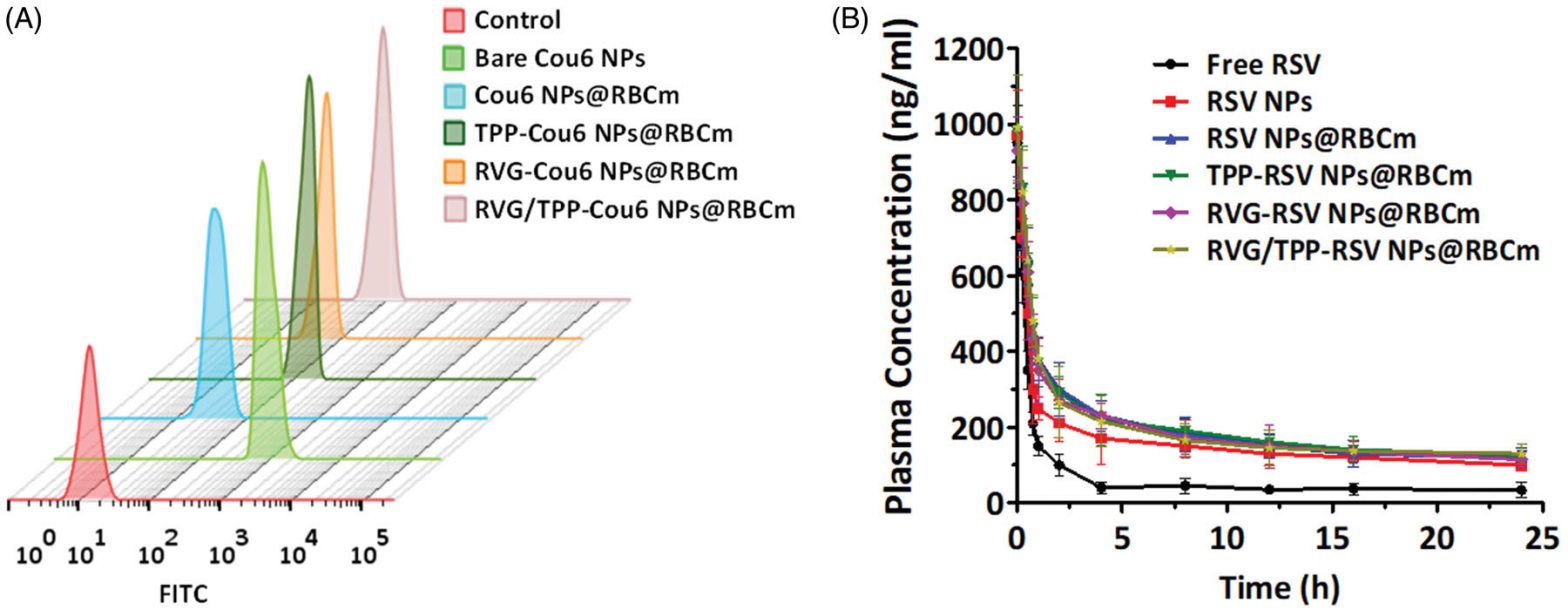
Long circulation feature. (A) FCM analysis of mouse peritoneal macrophages after incubation with various Cou6-tagged formulations. (B) Plasma RSV concentration-time profiles after i.v. injection of different formulations in rats. The data are presented as the means ± SD (*n* = 3).

The long-term circulation ability of nanosystems is crucial to achieve the target delivery. To verify whether RVG/TPP-RSV NPs@RBCm inherit the long circulation feature of the RBC, the *in vitro* antiphagocytosis assay was first conducted. Mouse peritoneal macrophages were utilized as a cell model to reckon the stealth power of novel biomimetic nanosystems. A less fluorescence level was visualized in macrophages treated with RVG/TPP-Cou6 NPs@RBCm and Cou6 NPs@RBCm than in the bare Cou6 NPs group (Figure 4(A)), indicating that the RBC membrane coating may effectively block nonspecific nanoparticle uptake by macrophages.

Accumulating evidence has suggested that the ability of RBCs to survive in macrophages is due to a collective contribution of the proteins and glycosyl groups on the cell membrane surface. Specifically, CD47, a well-documented protein marker firmly embedded in RBC membranes, can inhibit macrophage phagocytosis through interaction with the SIRP-α receptor (Anniss & Sparrow, 2002). To confirm the presence of CD47 on RVG29/TPP NPs@RBCm, western blotting analysis was conducted on a series of samples. As shown in Figure S2, the result unequivocally revealed the presence of CD47 on the surface of RVG29/TPP NPs@RBCm, indicating that RVG29/TPP NPs@RBCm retained the functions of RBC membrane-associated proteins. The results implied that RBC membranes could camouflage these bare NPs from being eliminated in circulation.

After a series of *in vitro* studies, a pharmacokinetics study was employed to investigate the *in vivo* behavior of RSV-loaded formulations (Figure 4(B) and Supplementary Table S2). It was found that all of the RSV-loaded novel biomimetic nanosystems (RSV NPs@RBCm, RVG-RSV NPs@RBCm, TPP-RSV NPs@RBCm and RVG/TPP-RSV NPs@RBCm) showed initially high blood circulating levels, while bare RSV NPs and free RSV were more quickly cleared up from the systemic circulation. The results confirmed the long circulation feature of biomimetic nanosystems *in vivo*, possibly due to the stealth property (Figure 4(A)) of the ‘outer shell’, as well as the sustained drug release profile (Figure 2(D)) of the ‘inner core’. Additionally, RSV-loaded nonmodified novel biomimetic nanosystems (RSV NPs@RBCm) and RSV-loaded ligand-modified novel biomimetic nanosystems (RVG-RSV NPs@RBCm, TPP-RSV NPs@RBCm and RVG/TPP-RSV NPs@RBCm) displayed resembling pharmacokinetic curves, and no significant difference was observed in the AUC, t_1/2_, MRT and CLz between them (*p* > .05). Thus, the post-insertion of RVG29 and/or TPP onto the surface of novel biomimetic nanosystems did not impair the long-circulation characteristic of RBC membranes. The results again emphasized the advantage of the postinsertion method in preparing ligand-modified novel biomimetic nanosystems.

### Neuronal mitochondria targeting

**Figure 5.**
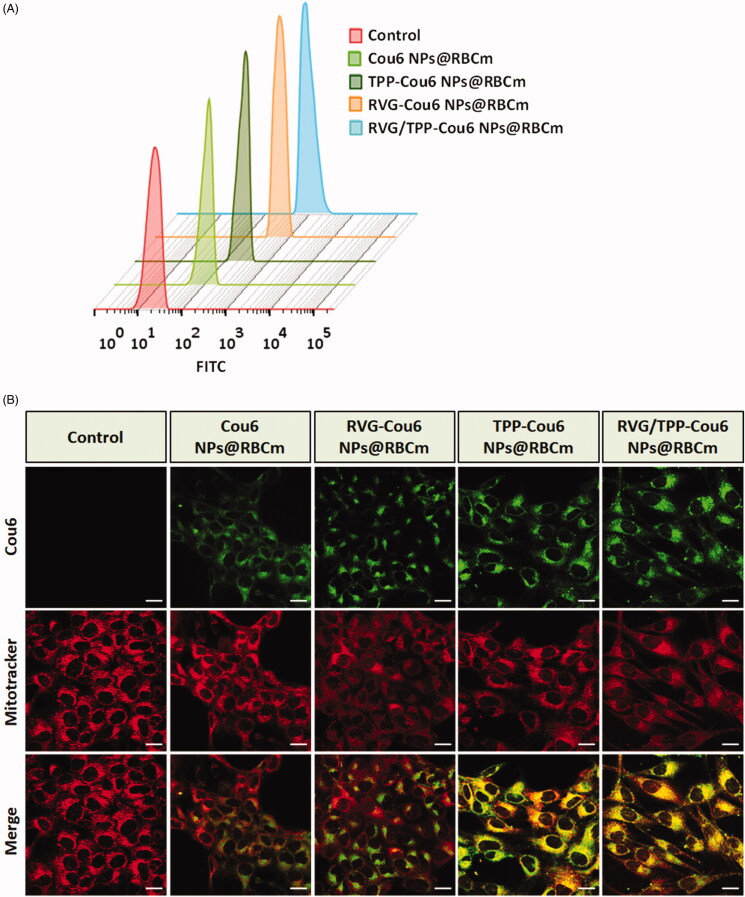
Neuronal cellular uptake and mitochondria targeting (A) FCM analysis of HT22 cells after incubation with different Cou6-tagged various formulations. (B) Colocalization of various Cou6-tagged formulations into mitochondria in HT22 cells. Cou6 (green) and MitoTracker for mitochondria staining (red) were recorded. Scale bars represent 20 μm.

One of the challenges in the treatment of ROS-induced mitochondrial dysfunction is the lack of a platform that delivers antioxidants to neuronal mitochondria. To assess the potential neuronal mitochondria targeting capability of ligand-modified novel biomimetic nanosystems, the neuronal cellular uptake of various Cou6-tagged formulations was conducted. As shown in Figure 5(A), the ligand-modified formulations (TPP-Cou6 NPs@RBCm, RVG-Cou6 NPs@RBCm and RVG/TPP-Cou6 NPs@RBCm) were significantly internalized into HT22 cells compared with nonmodified formulations (Cou6 NPs@RBCm), indicating that ligands could sufficiently improve entrance to the cells. Among all the tested formulations, RVG/TPP-Cou6 NPs@RBCm exhibited the maximum intracellular fluorescence intensity due to dual-mediated endocytosis. Although the exact uptake mechanism of RVG29 or TPP’s mediation has not been elucidated yet, it is possible that anchoring RVG29 and/or TPP on the novel biomimetic nanosystems changes the nanosystem’s lipophilicity and surface charge (Supplementary Table S1), enhancing its cell association and entrance into the cells by membrane partitioning (Biswas et al., 2012).

To explore whether these ligand-modified formulations targeted the mitochondria after successful internalization, mitochondrial colocalization study was conducted. Figure 5(B) illustrates images for the colocalization of novel biomimetic nanosystems into the mitochondria of HT22 cells after treatment with various Cou6-tagged formulations. Bright-yellow fluorescence and red (mitochondria) and green (Cou6-tagged formulations) fluorescence were used to indicate the colocalization of the Cou6-tagged novel biomimetic nanosystems into the mitochondria. According to the images, RVG/TPP-Cou6 NPs@RBCm exhibited a higher degree of green fluorescence and colocalization than TPP-Cou6 NPs@RBCm, a finding that was consistent with the data presented regarding neuronal cellular uptake. Although RVG-Cou6 NPs@RBCm can easily internalize into HT22 cells compared with Cou6 NPs@RBCm, the mitochondrial colocalization of RVG-Cou6 NPs@RBCm in HT22 cells were not ideal. The results indicated that RVG29 could efficiently improve neuron cell uptake but did not recognize and bind to the mitochondria; thus, RVG-Cou6 NPs@RBCm could not target mitochondria. Meanwhile, a higher degree of colocalization of fluorescence with the mitochondrial compartment was found for TPP-functionalized formulations (TPP-Cou6 NPs@RBCm and RVG/TPP-Cou6 NPs@RBCm) compared with that for Cou6 NPs@RBCm and RVG-Cou6 NPs@RBCm, suggesting a role for TPP. Based on these results, we conclude that the TPP moiety potentiates the neuronal mitochondria-targeting ability of novel biomimetic nanosystems.

### Transport across the BBB

**Figure 6.**
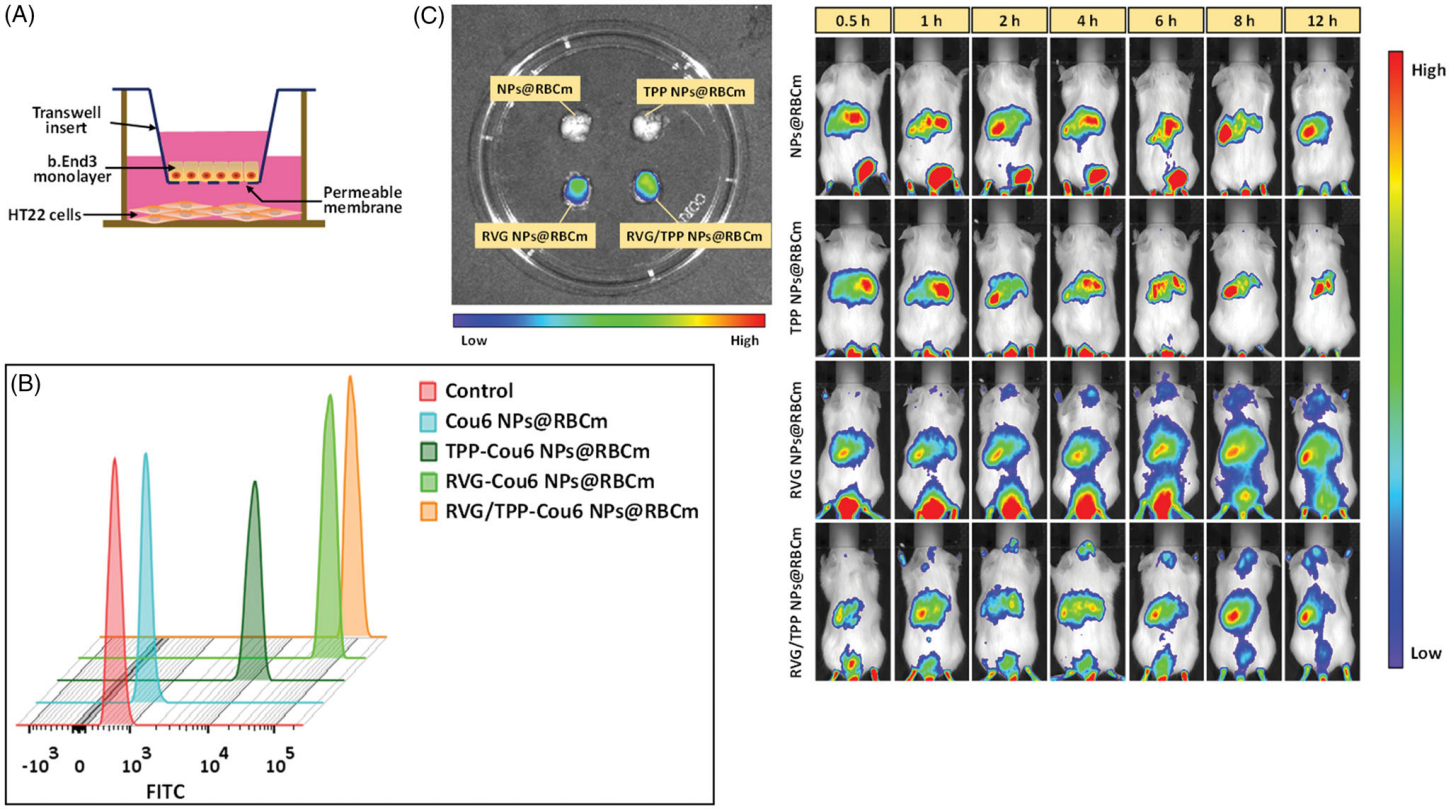
Transport across the BBB of novel biomimetic nanosystems. (A) Schematic diagram of *in vitro* BBB model. (B) FCM analysis of HT22 cells treated with various Cou6-tagged formulations after crossing the BBB-HT22 cells co-culture *in vitro* model. (C) *In vivo* brain-targeting ability. Biodistribution of DIR contained in various formulations determined by IVIS Lumina II.

The ability to cross the BBB is one of the major prerequisites for nanocarriers to mediate neuronal mitochondria-targeting drug delivery. After the neuronal mitochondria-targeting ability of TPP-modified formulations was verified, BBB permeability studies were conducted. First, a BBB/HT22 cell co-culture *in vitro* model (Figure 6(A)) was constructed to assay the targeting ability of these Cou6-tagged novel biomimetic nanosystems. As shown in Figure 6(B), the fluorescence intensity remains a very low level in the lower chamber of HT22 cells after incubating with Cou6 NPs@RBCm for 12 h, indicating that it could not effectively pass through the BBB. The difference in the cellular uptake in the co-culture model between Cou6 NPs@RBCm and TPP-Cou6 NPs@RBCm was not remarkble, implying TPP’s lack of penetrating BBB ability, whereas the relative high uptake in TPP-Cou6 NPs@RBCm-treated HT22 cells was derived from the promoted entry of nanosystems into cells via TPP (Figure 5(A)). By contrast, moderate fluorescence was found in the cells with RVG-Cou6 NPs@RBCm treatment, suggesting that the RVG29 mono-modified formulations could pass through the BBB and internalize into HT22 cells. The results again emphasize the advantage of RVG29, which can efficiently cross the BBB into neuron cells with high selectivity (Park et al., 2015). As we expected, a significant signal was observed in the bottom chamber of HT22 cells with RVG/TPP-Cou6 NPs@RBCm during the experimental period, indicating that the dual-modified formulations could efficiently cross the BBB and sufficiently promote uptake in HT22 cells under the synergistic effect of RVG29 and TPP.

To verify the actual penetrating BBB ability of RVG/TPP NPs@RBCm, *in vivo* imaging was performed on mice using various DIR-tagged biomimetic formulations by tail vein injection. Based on whole-body imaging (Figure 6(C)), the brain accumulation of DIR-tagged NPs@RBCm and DIR-tagged TPP NPs@RBCm did not occur. By contrast, high accumulation of DIR-tagged RVG NPs@RBCm was detected in the brain at 0.5 h after injection. Importantly, the most intense distribution in the brain was displayed in the DIR-tagged RVG/TPP NPs@RBCm-treated mice and was further confirmed by the strongest fluorescence identified in the isolated brain. This phenomenon indicated that the introduction of RVG29 induced brain targeting in the hydrophobic probe and the incorporation of TPP, further enhancing its accumulation in neurons. The *in vivo* imaging results agreed with the results of the *in vitro* coculture model shown in Figure 6(B). These initial data demonstrated that RVG29-functionalized formulations (RVG NPs@RBCm and RVG/TPP NPs@RBCm) could pass through the BBB efficiently. Overall, these above results (Figures 5 and 6) demonstrated that the dual-modified novel biomimetic nanosystems could not only penetrate the BBB but also target the neuron cells and further localize in the mitochondria under the synergistic effect of RVG29 and TPP.

### *In vitro* antioxidative stress effect

**Figure 7.**
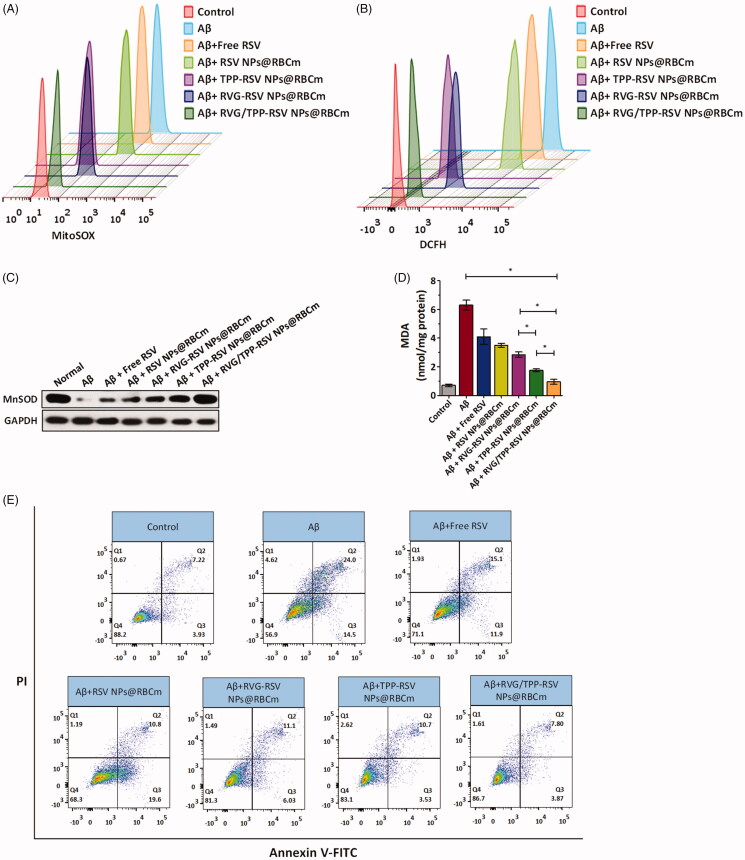
Therapeutic effect of RSV-loaded novel biomimetic nanosystems *in vitro*. FCM analysis of (A) mitochondrial ROS accumulation obtained by MitoSOX and (B) intracellular H_2_O_2_ level by CM-H_2_DCFDA in Aβ_23–35_-damaged HT22 cells after treatment with different RSV-loaded formulations. Changes of (C) MnSOD and (D) MDA levels in Aβ_23–35_-damaged HT22 cells after treatment with different RSV-loaded formulations. (E) Apoptosis results of Aβ_23–35_-damaged HT22 cells after treatment with different RSV-loaded formulations. The data are presented as the means ± SD (*n* = 3). * indicates *p* < 0.05.

The protection of mitochondria against oxidative stress from ROS is therapeutically beneficial to AD (Wang & Chen, 2016). The protective role of resveratrol (RSV) in neuron cells, along with the potent antioxidant and free radical scavenging activity, has been widely studied (Khanduja & Bhardwaj, 2003; Jin et al., 2016). Accordingly, we studied the mitochondria as a therapeutic target against AD and evaluated the *in vitro* antioxidative stress effects using RSV-loaded novel biomimetic nanosystems. The scavenging capacity of RSV-loaded formulations for mitochondrial ROS (·O_2_^−^) was measured by FCM (Figure 7(A)). We found that Aβ_25–35_ induced the upregulation of the mitochondrial ROS level, consistent with the previous studies (Zhang et al., 2013). Interestingly, RSV-loaded, TPP-functionalized formulations (TPP-RSV NPs@RBCm and RVG/TPP-RSV NPs@RBCm) could more strongly inhibit mitochondrial ROS in Aβ-treated HT22 neuronal cells after targeting mitochondria than other groups, a finding that could be attributed to TPP delivery. As we expected, RVG/TPP-RSV NPs@RBCm presented the strongest inhibition of mitochondrial ROS. By contrast, the inhibitory capability of RVG-RSV NPs@RBCm and RSV NPs@RBCm was not ideal, likely attributed to their lack of mitochondria-targeting ability. These results were consistent with the mitochondrial colocalization (Figure 5(B)) described above.

Intracellular H_2_O_2_ is a toxic byproduct of aerobic metabolism, which is related signal pathways and plays a crucial role in cellular apoptosis. In addition, MnSOD and MDA are well known biomarkers of oxidative stress. SOD plays important roles in antioxidant defense, and its depletion is one of the crucial clues in cellular toxicity. Among SODs, MnSOD is the main SOD species in mitochondria (Tang et al., 2014). The increased MDA is an important indicator of lipid peroxidation, which is toxic to cells. These oxidative stress-related biomarkers were measured to further determine whether these RSV-loaded formulations reduced oxidative stress from ROS in the Aβ-treated HT22 neuronal cells (Figure 7(B–D)). Because TPP-functionalized formulations are predominantly targeted to mitochondria, TPP-RSV NPs@RBCm cause more powerful oxidative stress reduction than RSV NPs@RBCm and RVG-RSV NPs@RBCm. Additionally, compared with other groups, RVG/TPP-RSV NPs@RBCm exhibited the greatest oxidative-stress reduction effect in the Aβ-treated HT22 cells, a finding that was consistent with the data presented regarding cellular uptake (Figure 5(A)) and mitochondria targeting (Figure 5(B)).

Mitochondrial ROS accumulation is known to be a major cause of neuronal apoptosis, and ROS clearance can benefit the viability to rescue mitochondrial pathology (Marrache & Dhar, 2012). Because TPP-functionalized formulations containing RSV can inhibit mitochondrial ROS, we further evaluated whether they affected cell apoptosis. As shown in Figure 7(E), the apoptosis of HT22 cells was increased to 38.5% when the cells were stimulated with Aβ_25–35_. Consistent with these findings in mitochondrial ROS measurement, RVG/TPP-RSV NPs@RBCm treatment decreased the cell apoptosis rate to 11.67%, much more effective than TPP-RSV NPs@RBCm (14.23% apoptosis), RVG-RSV NPs@RBCm (17.13% apoptosis), RSV NPs@RBCm (30.4% apoptosis) and free RSV treatment (27.00% apoptosis), respectively. The results demonstrated that RVG/TPP-RSV NPs@RBCm can effectively protect HT22 neurons from apoptosis.

### *In vivo* therapeutic effect

**Figure 8.**
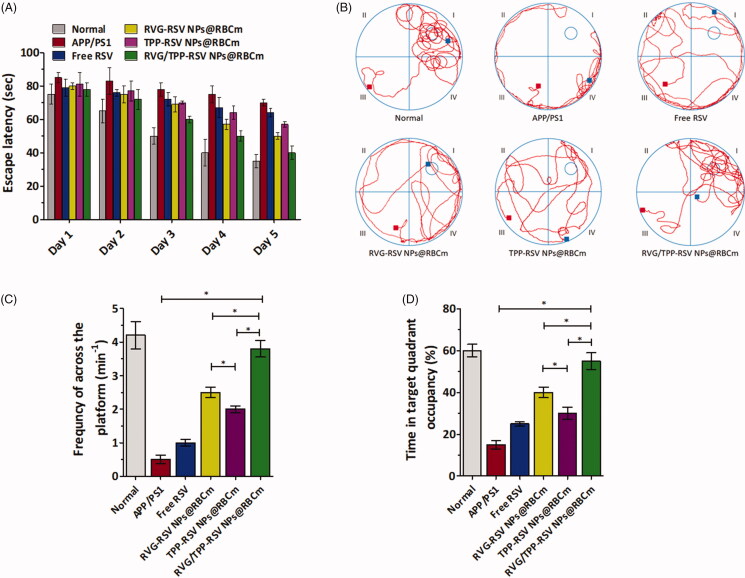
*In vivo* evaluation of RSV-loaded novel biomimetic nanosystems therapy. (A) Escape latency time of each group. (B) Representative swimming path tracings of different groups. (C) The frequncy of platform crossing at the final day with the platform removed. (D) Relative time spent on the target quadrant. The data are presented as the means ± SD (*n* = 3). * indicates *p* < 0.05.

With advantages in suitable physiochemical properties, the long circulation feature, safety, multitargeting abilities and *in vitro* antioxidative stress effect, we subsequently evaluated the effect of RVG/TPP-RSV NPs@RBCm in alleviating the progression of AD pathology. The MWM test was used to detect whether the RSV-loaded dual-modified novel biomimetic nanosystems could improve spatial learning ability in APP/PS1 mice. As shown in Figure 8(A–D), saline treatment APP/PS1 group showed significant learning deficits. Due to the weak BBB-penetrating efficiency, APP/PS1 mice treated with free RSV or TPP-RSV NPs@RBCm showed slightly improved learning ability. Owing to the high BBB penetrating efficacy, APP/PS1 mice moderately alleviated defects following the administration of RVG-RSV NPs@RBCm. Furthermore, RVG/TPP-RSV NPs@RBCm significantly improved the cognitive ability of APP/PS1 mice by shortening the escape latency (Figure 8(A)), restoring the spatial learning ability (Figure 8(B)), increasing the frequency of across the platform (Figure 8(C)), and prolonging the time spent in a targeted quadrant after removing the platform (Figure 8(D)), compared with that of other groups. The above results imply that the anti-AD effect of RVG/TPP-RSV NPs@RBCm is stronger than that of other preparations in the model animals.

**Figure 9.**
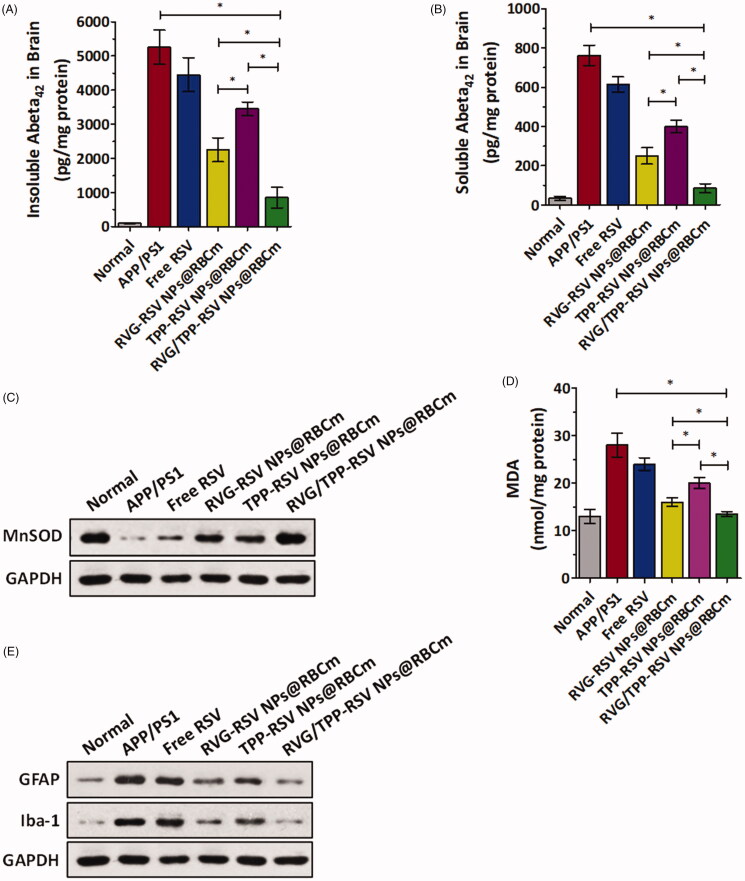
Neuroprotective effects *in vivo*. (A) Insoluble and (B) soluble Aβ_1–42_ in the brain. The data are presented as the means ± SD (*n* = 3). *p < 0.05 with respect to the APP/PS1 mice. Changes of (C) MnSOD and (D) MDA levels in the hippocampal area of APP/PS1 mice after i.v. injection with different RSV-loaded formulations. (E) Changes of GFAP and Iba-1 protein levels in the hippocampal area of APP/PS1 mice after i.v. injection with different RSV-loaded formulations.

Aβ plaque is one of the key markers of AD. To investigate the inhibitory effect of the RSV-loaded novel biomimetic nanosystems on Aβ in vivo, the amount of soluble and insoluble Aβ_1-42_ was measured by enzyme-linked immunosorbent assay (ELISA) in APP/PS1 mice brain homogenate (Figure 9(A,B)). The amounts of soluble and insoluble Aβ_1–42_ in the brain homogenates of the RVG/TPP-RSV NPs@RBCm treatment group decreased significantly in APP/PS1 model mice compared with other formulations. The results confirmed that the observed decreased levels of Aβ in brain were accompanied with learning and memory improvements (Figure 8). Because oxidative stress could cause neuronal cell structural and functional damage, improving the oxidative stress in brain may protect neurons (Verdin et al., 2010). Previous studies have also shown that ROS-caused oxidative stress not only acts as an inducer but also possesses a sustaining factor in AD (Swerdlow & Khan, 2004). Next, the levels of MnSOD and MDA were measured to determine whether RSV-loaded novel biomimetic nanosystems could protect against oxidative stress from ROS in the hippocampus of APP/PS1 mice (Figure 9(C,D)). The results of these biomarkers assays showed that the RVG/TPP-RSV NPs@RBCm effectively restored the decreased MnSOD levels and reduced the lipid peroxidation damage caused by oxidative stress in the hippocampus. The tendency found above agreed with the finding of the *in vitro* antioxidative stress effect (Figure 7(C,D)).

Recent studies have demonstrated that microglia and astrocytes are the principal immune cells of the brain and play important roles in the development of neurodegenerative disease, including AD (Mhatre et al., 2015). Elimination of microglial and astrocytic activation might be therapeutically beneficial to AD (Asai et al., 2015). Therefore, we further analyzed the effect of RVG/TPP-RSV NPs@RBCm on inflammation related to astrocytic and microglial activation after the MWM test. As shown in Figure 9(E), glial fibrillary acidic protein (GFAP, a biomarker of astrocytes) and ionized calcium binding adaptor molecule-1 (Iba-1, a biomarker of microglia) demonstrated significant higher levels in the APP/PS1 mice, indicative of reactive gliosis. As expected, both GFAP and Iba-1 protein levels were significantly decreased in the hippocampus of the APP/PS1 mice treated with RVG/TPP-RSV NPs@RBCm compared with other formulations. The results suggested that RVG/TPP-RSV NPs@RBCm could mitigate brain inflammation by improving mitochondrial oxidative stress in APP/PS1 mice. The above results suggested that the mitochondrial antioxidative ability of RVG/TPP-RSV NPs@RBCm contributed to protecting neurons and, consequently, rescues the memory deficits of APP/PS1 mice.

Overall, the above findings indicate that RVG/TPP-RSV NPs@RBCm exert stronger anti-AD activity than other formulations in both Aβ-damaged neuron cells and AD model mice. These *in vitro* and *in vivo* results (Figures 7–9) are consistent with previous findings, suggesting the superiority of RVG/TPP NPs@RBCm investigated for the physiochemical properties (Figure 2), safety (Figure 3), long circulation feature (Figure 4 and Supplementary Figure S2), mitochondria targeting (Figure 5) and BBB permeability (Figure 6). The RSV-loaded dual-modified novel biomimetic nanosystems are a potential therapeutic candidate to treat neuronal mitochondrial ROS-induced damage in AD.

## Conclusions

In this study, we constructed a novel biomimetic drug delivery nanosystem with dual modifications (RVG/TPP NPs@RBCm) that can safely and specifically deliver antioxidants into neuronal mitochondria upon intravenous administration for the treatment of AD. The suitable physicochemical properties of the ‘inner core’ and unique biological functions of the ‘outer shell’ jointly contribute to the good biocompatibility and long-term circulation of RVG/TPP NPs@RBCm. With the assistance of the anchored RVG29 and TPP on the ‘outer shell’, the dual-modified novel biomimetic nanosystems could efficiently cross the BBB and specifically bind to neurons, subsequently reaching the mitochondria. Although preliminary, the research data of this work collectively suggest that RVG/TPP NPs@RBCm might provide a promising intravenous mitochondria-targeted delivery nanosystem for AD treatment. In a future study, we will perform *in vitro* and *in vivo* studies examining the targeted delivery mechanism and further explore the application of RVG/TPP NPs@RBCm in neurodegenerative disease therapy.

## Supplementary Material

Supplemental Material

## References

[CIT0001] Anniss AM, Sparrow RL. (2002). Expression of CD47 (integrin-associated protein) decreases on red-blood cells during storage. Transfus Apher Sci 27:233–8. 12509218 10.1016/s1473-0502(02)00070-8

[CIT0002] Asai H, Ikezu S, Tsunoda S, et al. (2015). Depletion of microglia and inhibition of exosome synthesis halt tau propagation. Nat Neurosci 18:1584–93. 26436904 10.1038/nn.4132 PMC4694577

[CIT0003] Ballard C, Gauthier S, Corbett A, et al. (2011). Alzheimer’s disease. Lancet 377:1019–31. 21371747 10.1016/S0140-6736(10)61349-9

[CIT0004] Biswas S, Dodwadkar NS, Deshpande PP, Torchilin VP. (2012). Liposomes loaded with paclitaxel and modified with novel triphenylphosphonium-PEG-PE conjugate possess low toxicity, target mitochondria and demonstrate enhanced antitumor effects in vitro and in vivo. J Control Release 159:393–402. 22286008 10.1016/j.jconrel.2012.01.009 PMC3348446

[CIT0005] Caldwell CC, Yao J, Brinton RD. (2015). Targeting the prodromal stage of Alzheimer’s disease: bioenergetic and mitochondrial opportunities. Neurotherapeutics 12:66–80. 25534394 10.1007/s13311-014-0324-8 PMC4322082

[CIT0006] Careri M, Corradini C, Elviri L, et al. (2003). Direct HPLC analysis of quercetin and trans-resveratrol in red wine, grape, and winemaking byproducts. J Agric Food Chem 51:5226–31. 12926863 10.1021/jf034149g

[CIT0007] Celia C, Trapasso E, Cosco D, et al. (2009). Turbiscan lab (R) expert analysis of the stability of ethosomes (R) and ultradeformable liposomes containing a bilayer fluidizing agent. Colloid Surface B 72:155–60. 10.1016/j.colsurfb.2009.03.007 19376689

[CIT0008] Chai ZL, Ran DN, Lu LW, et al. (2019). Ligand-modified cell membrane enables the targeted delivery of drug nanocrystals to glioma. Acs Nano 13:5591–601. 31070352 10.1021/acsnano.9b00661

[CIT0009] Cui GH, Guo HD, Li H, et al. (2019). RVG-modified exosomes derived from mesenchymal stem cells rescue memory deficits by regulating inflammatory responses in a mouse model of Alzheimer’s disease. Immun Ageing 16:10. 31114624 10.1186/s12979-019-0150-2 PMC6515654

[CIT0010] Dodge JT, Hanahan DJ, Mitchell C. (1963). Preparation and chemical characteristics of hemoglobin-free ghosts of human erythrocytes. Arch Biochem Biophys 100:119–30. 14028302 10.1016/0003-9861(63)90042-0

[CIT0011] Doody RS, Raman R, Farlow M, et al. (2013). A phase 3 trial of semagacestat for treatment of Alzheimer’s disease. N Engl J Med 369:341–50. 23883379 10.1056/NEJMoa1210951

[CIT0012] Doody RS, Thomas RG, Farlow M, et al. (2014). Phase 3 trials of solanezumab for mild-to-moderate Alzheimer’s disease. N Engl J Med 370:311–21. 24450890 10.1056/NEJMoa1312889

[CIT0013] Fang RNH, Hu CMJ, Chen KNH, et al. (2013). Lipid-insertion enables targeting functionalization of erythrocyte membrane-cloaked nanoparticles. Nanoscale 5:8884–8. 23907698 10.1039/c3nr03064d PMC3831007

[CIT0014] Fang RH, Jiang Y, Fang JC, Zhang LF. (2017). Cell membrane-derived nanomaterials for biomedical applications. Biomaterials 128:69–83. 28292726 10.1016/j.biomaterials.2017.02.041 PMC5417338

[CIT0015] Fu SY, Liang M, Wang YL, Cui L, et al. (2019). Dual-modified novel biomimetic nanocarriers improve targeting and therapeutic efficacy in glioma. ACS Appl Mater Interfaces 11:1841–54. 30582685 10.1021/acsami.8b18664

[CIT0016] Fuhrmann T, Ghosh M, Otero A, et al. (2015). Peptide-functionalized polymeric nanoparticles for active targeting of damaged tissue in animals with experimental autoimmune encephalomyelitis. Neurosci Lett 602:126–32. 26141613 10.1016/j.neulet.2015.06.049

[CIT0017] Hardy J, Allsop D. (1991). Amyloid deposition as the central event in the etiology of alzheimers-disease. Trends Pharmacol Sci 12:383–8. 1763432 10.1016/0165-6147(91)90609-v

[CIT0018] He H, Chen XJ, Wang GJ, et al. (2006). High-performance liquid chromatography spectrometric analysis of trans-resveratrol in rat plasma. J Chromatogr B 832:177–80. 10.1016/j.jchromb.2005.12.021 16446129

[CIT0019] Jack CR, Knopman DS, Chetelat G, et al. (2016). Suspected non-Alzheimer disease pathophysiology - concept and controversy. Nat Rev Neurol 12:117–24. 26782335 10.1038/nrneurol.2015.251 PMC4784257

[CIT0020] Jin J, Shi F, Li QW, et al. (2016). Evaluation of free radical scavenging capacity and antioxidative damage effect of resveratrol-nanostructured lipid carriers. Proc Spie 9722, Colloidal Nanoparticles for Biomedical Applications XI, 97221D; 2016 April 22.

[CIT0021] Kerr JS, Adriaanse BA, Greig NH, et al. (2017). Mitophagy and Alzheimer’s disease: cellular and molecular mechanisms. Trends Neurosci 40:151–66. 28190529 10.1016/j.tins.2017.01.002 PMC5341618

[CIT0022] Khanduja KL, Bhardwaj A. (2003). Stable free radical scavenging and antiperoxidative properties of resveratrol compared in vitro with some other bioflavonoids. Indian J Biochem Bio 40:416–22. 22900369

[CIT0023] Kwon HJ, Cha MY, Kim D, Kim DK, et al. (2016). Mitochondria-targeting ceria nanoparticles as antioxidants for Alzheimer’s disease. ACS Nano 10:2860–70. 26844592 10.1021/acsnano.5b08045

[CIT0024] Lin MT, Beal MF. (2006). Mitochondrial dysfunction and oxidative stress in neurodegenerative diseases. Nature 443:787–95. 17051205 10.1038/nature05292

[CIT0025] Lovell MA, Xiong SL, Xie CS, et al. (2005). Induction of hyperphosphorylated tau in primary rat cortical neuron cultures mediated by oxidative stress and glycogen synthase kinase-3. JAD 6:659–71. 10.3233/jad-2004-6610 15665406

[CIT0026] Marrache S, Dhar S. (2012). Engineering of blended nanoparticle platform for delivery of mitochondria-acting therapeutics. Proc Natl Acad Sci USA 109:16288–93. 22991470 10.1073/pnas.1210096109 PMC3479596

[CIT0027] Mhatre SD, Tsai CA, Rubin AJ, et al. (2015). Microglial malfunction: the third rail in the development of Alzheimer’s disease. Trends Neurosci 38:621–36. 26442696 10.1016/j.tins.2015.08.006 PMC4670239

[CIT0028] Omidi Y, Campbell L, Barar J, et al. (2003). Evaluation of the immortalised mouse brain capillary endothelial cell line, b.End3, as an in vitro blood–brain barrier model for drug uptake and transport studies. Brain Res 990:95–112. 14568334 10.1016/s0006-8993(03)03443-7

[CIT0029] Park TE, Singh B, Li H, et al. (2015). Enhanced BBB permeability of osmotically active poly(mannitol-co-PEI) modified with rabies virus glycoprotein via selective stimulation of caveolar endocytosis for RNAi therapeutics in Alzheimer’s disease. Biomaterials 38:61–71. 25457984 10.1016/j.biomaterials.2014.10.068

[CIT0030] Salloway S, Sperling R, Fox NC, et al. (2014). Two phase 3 trials of bapineuzumab in mild-to-moderate Alzheimer’s disease. N Engl J Med 370:322–33. 24450891 10.1056/NEJMoa1304839 PMC4159618

[CIT0031] Sawda C, Moussa C, Turner RS. (2017). Resveratrol for Alzheimer’s disease. Ann NY Acad Sci 1403:142–9. 28815614 10.1111/nyas.13431 PMC5664214

[CIT0032] Schonhegrad MA, Holt PG. (1981). Improved method for the isolation of purified mouse peritoneal-macrophages. J Immunol Methods 43:169–73. 7264322 10.1016/0022-1759(81)90020-x

[CIT0033] Selfridge JE, E LZ, Lu JH, Swerdlow RH. (2013). Role of mitochondrial homeostasis and dynamics in Alzheimer’s disease. Neurobiol Dis 51:3–12. 22266017 10.1016/j.nbd.2011.12.057 PMC3337963

[CIT0034] Shafiei SS, Guerrero-Munoz MJ, Castillo-Carranza DL. (2017). Tau oligomers: cytotoxicity, propagation, and mitochondrial damage. Front Aging Neurosci 9:83. 28420982 10.3389/fnagi.2017.00083 PMC5378766

[CIT0035] Singh M, Arseneault M, Sanderson T, et al. (2008). Challenges for research on polyphenols from foods in Alzheimer’s disease: bioavailability, metabolism, and cellular and molecular mechanisms. J Agric Food Chem 56:4855–73. 18557624 10.1021/jf0735073

[CIT0036] Swerdlow RH, Khan SM. (2004). A “mitochondrial cascade hypthesis” for sporadic Alzheimer’s disease. Med Hypotheses 63:8–20. 15193340 10.1016/j.mehy.2003.12.045

[CIT0037] Tang XQ, Luo YX, Chen HZ, Liu DP. (2014). Mitochondria, endothelial cell function, and vascular diseases. Front Physiol 5:175. 24834056 10.3389/fphys.2014.00175 PMC4018556

[CIT0038] Verdin E, Hirschey MD, Finley LWS, Haigis MC. (2010). Sirtuin regulation of mitochondria: energy production, apoptosis, and signaling. Trends Biochem Sci 35:669–75. 20863707 10.1016/j.tibs.2010.07.003 PMC2992946

[CIT0039] Walle T, Hsieh F, DeLegge MH, et al. (2004). High absorption but very low bioavailability of oral resveratrol in humans. Drug Metab Dispos 32:1377–82. 15333514 10.1124/dmd.104.000885

[CIT0040] Wang Y, Catana F, Yang YN, et al. (2002). An LC-MS method for analyzing total resveratrol in grape juice, cranberry juice, and in wine. J Agric Food Chem 50:431–5. 11804508 10.1021/jf010812u

[CIT0041] Wang J, Chen GJ. (2016). Mitochondria as a therapeutic target in Alzheimer’s disease. Genes Dis 3:220–7. 30258891 10.1016/j.gendis.2016.05.001 PMC6150105

[CIT0042] Wang Q, Liu TT, Fu Y, et al. (2010). Vanadium compounds discriminate hepatoma and normal hepatic cells by differential regulation of reactive oxygen species. J Biol Inorg Chem 15:1087–97. 20443032 10.1007/s00775-010-0668-4

[CIT0043] Wang Y, Wang N, Cai B, et al. (2015). In vitro model of the blood–brain barrier established by co-culture of primary cerebral microvascular endothelial and astrocyte cells. Neural Regen Res 10:2011–7. 26889191 10.4103/1673-5374.172320 PMC4730827

[CIT0044] Yang H. (2010). Nanoparticle-mediated brain-specific drug delivery, imaging, and diagnosis. Pharm Res 27:1759–71. 20593303 10.1007/s11095-010-0141-7 PMC2933363

[CIT0045] Yao L, Gu X, Song QX, et al. (2016). Nanoformulated alpha-mangostin ameliorates Alzheimer’s disease neuropathology by elevating LDLR expression and accelerating amyloid-beta clearance. J Control Release 226:1–14. 26836197 10.1016/j.jconrel.2016.01.055

[CIT0046] Yao J, Irwin RW, Zhao LQ, et al. (2009). Mitochondrial bioenergetic deficit precedes Alzheimer’s pathology in female mouse model of Alzheimer’s disease. Proc Natl Acad Sci USA 106:14670–5. 19667196 10.1073/pnas.0903563106 PMC2732886

[CIT0047] Yun J, Finkel T. (2014). Mitohormesis. Cell Metab 19:757–66. 24561260 10.1016/j.cmet.2014.01.011 PMC4016106

[CIT0048] Zhang GL, Zhang WG, Du Y, et al. (2013). Edaravone ameliorates oxidative damage associated with a beta 25-35 treatment in PC12 cells. J Mol Neurosci 50:494–503. 23393032 10.1007/s12031-013-9973-z

[CIT0049] Zhao ZX, Gao SY, Wang JC, et al. (2012). Self-assembly nanomicelles based on cationic mPEG-PLA-b-polyarginine(R-15) triblock copolymer for siRNA delivery. Biomaterials 33:6793–807. 22721724 10.1016/j.biomaterials.2012.05.067

