# Supplementary material for: Neuronal mitochondria-targeted therapy for Alzheimer’s disease by systemic delivery of resveratrol using dual-modified novel biomimetic nanosystems
Source: Drug Deliv. 2020 Mar 31;27(1):502–18. doi: 10.1080/10717544.2020.1745328 (PMC7170363; doi:10.1080/10717544.2020.1745328)
Supplement: Supplemental Material [file IDRD_A_1745328_SM4312.docx]

Supporting Information

**Neuronal Mitochondria-Targeted Therapy for Alzheimer’s Disease by Systemic Delivery of Resveratrol using Dual-Modified Novel Biomimetic Nanosystems**

Yang Han^a^, Xiaoyang Chu^c^, Lin Cui^b^, Shiyao Fu^b^, Chunsheng Gao^b^, Yi Li^b, *^, Baoshan Sun^d, e, *^

*^a^ School of Traditional Chinese Medicine, Shenyang Pharmaceutical University, Shenyang 110016, PR China*

*^b^State Key Laboratory of Toxicology and Medical Countermeasures, Beijing Institute of Pharmacology and Toxicology, Beijing 100850, PR China*

*^c^The Fifth Medical Center of PLA General Hospital，Beijing 100071, China*

*^d^School of Functional Food and Wine, Shenyang Pharmaceutical University, Shenyang 110016, PR China*

*^e^Instituto National de Investigação Agrária e Veterinária, I.P., Pólo Dois Portos, Quinta da Almoinha, Dois Portos 2565-191, Portugal*

*Corresponding Author: liyi_evening@pku.edu.cn; sun.baoshan@iniav.pt

**Fig. S1.** MALDI-TOF mass spectra of DSPE-PEG_2000_-TPP (A) and H^1^-NMR spectra of DSPE-PEG_2000_-RVG29 (B).

**Fig. S2.** Representative western blot analysis of CD47 in bare NLC, RBC lysate, RBC membrane and RVG/TPP NPs@RBCm.


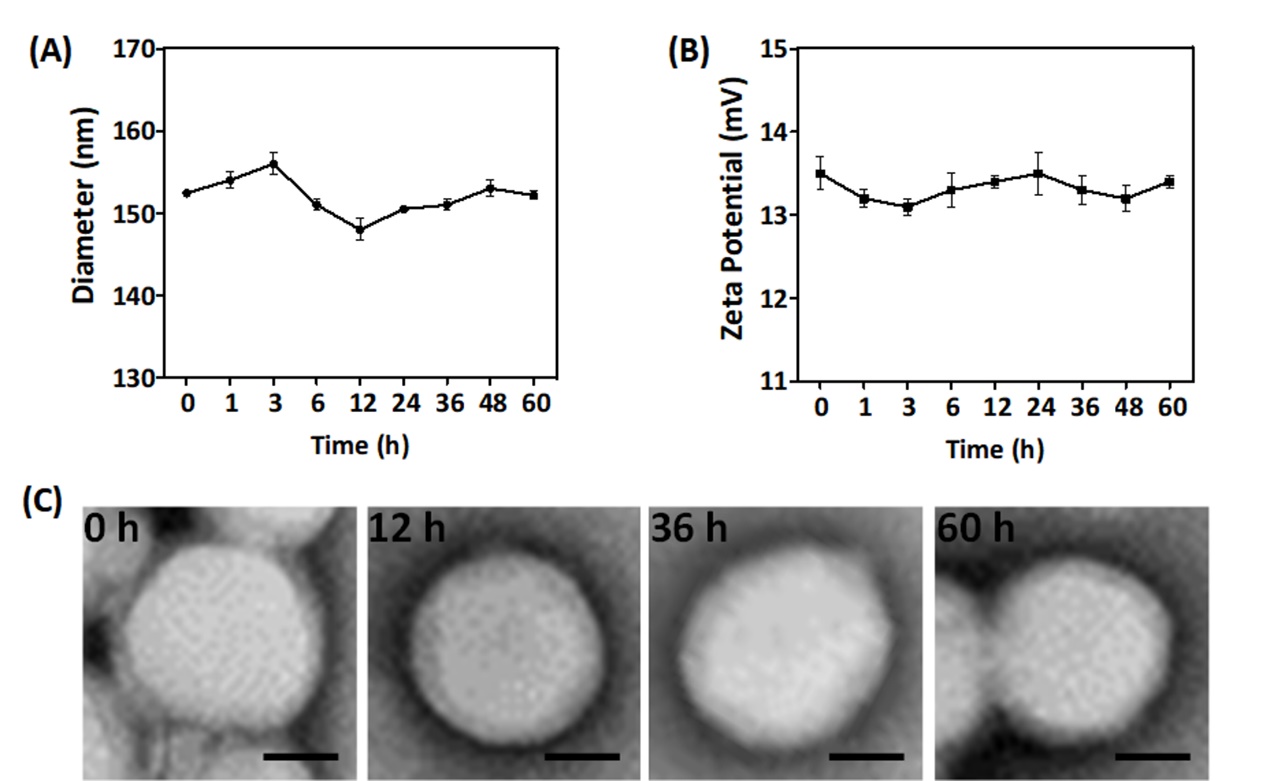


**Fig. S3.** Stability characterization. Partical diameter (A) and zeta potential (B) stability of RVG/TPP NPs@RBCm. The data are presented as the means ± SD (n = 3). (C) TEM images of RVG/TPP NPs@RBCm. Scale bar = 50 nm.

**Table S1.** Characteristics of the biomimetic nanoparticles

| Sample ID | Diameter (nm) | PDI | Zeta-potential (mV) |
| --- | --- | --- | --- |
| RSV NPs | 136.25± 0.46 | 0.094 ± 0.017 | -15.6 ± 0.8 |
| RSV NPs@RBCm | 148.51± 0.94 | 0.071 ± 0.010 | -23.6 ± 0.4 |
| RVG-RSV NPs@RBCm | 151.34 ± 0.72 | 0.083 ± 0.020 | -21.7 ± 0.7 |
| TPP-RSV NPs@RBCm | 150.71± 0.31 | 0.091 ± 0.014 | 17.3 ± 0.1 |
| RVG/TPP-RSV NPs@RBCm | 152.42± 0.37 | 0.088 ± 0.011 | 13.5 ± 0.2 |

The data are expressed as the mean ± SD for three different preparations (n = 3).

**Table S2.** Pharmacokinetic parameters of RSV-loaded biomimetic nanoparticles

| Sample ID | AUC_0→t_ (mg/L×h) | AUC_0→∞_ (mg/L×h) | t_1/2_  (h) | MRT_0→t_  (h) | CLz  (L/h/kg) |
| --- | --- | --- | --- | --- | --- |
| Free RSV | 1.47 ± 0.35 | 2.25 ± 1.31 | 2.37 ± 0.09 | 1.21 ± 0.26 | 1.73 ± 0.72 |
| RSV NPs | 2.84 ± 0.48 | 3.37 ± 0.79 | 4.55 ± 0.29 | 3.95 ± 0.12 | 1.10 ± 0.06 |
| RSV NPs@RBCm | 4.42 ±0.61 | 5.97 ± 1.12 | 8.27 ± 0.11 | 6.89 ± 0.15 | 0.46 ± 0.16 |
| TPP-RSV NPs@RBCm | 4.78 ± 0.33 | 6.32 ± 0.81 | 8.56 ± 0.03 | 6.39 ± 0.39 | 0.44 ± 0.07 |
| RVG-RSV NPs@RBCm | 4.55 ± 0.62 | 6.14 ± 0.36 | 8.83 ± 0.12 | 6.23 ± 0.57 | 0.49 ± 0.02 |
| RVG/TPP-RSV NPs@RBCm | 4.33 ± 0.40 | 5.69 ± 0.92 | 8.06 ± 0.39 | 6.24 ± 0.64 | 0.42 ± 0.09 |

AUC_0→t_: the area under the blood concentration time curve from 0 to the last selected time point; AUC_0→∞_: the area under the blood concentration time curve during the period from 0 to infinity; t_1/2_: elimination half-life; MRT: mean residence time; CLz: total clearance. The data are expressed as the mean ± SD for three different preparations (n = 3).
